# ﻿Four new species of *Sphaeroderma* Stephens (Coleoptera, Chrysomelidae, Galerucinae, Alticini) from Taiwan, with discussion on genus boundaries based on *S.flavonotatum* Chûjô and *S.jungchani* sp. nov.

**DOI:** 10.3897/zookeys.1185.112099

**Published:** 2023-11-27

**Authors:** Chi-Feng Lee

**Affiliations:** 1 Applied Zoology Division, Taiwan Agricultural Research Institute, Taichung 413, Taiwan Applied Zoology Division, Taiwan Agricultural Research Institute Taichung Taiwan

**Keywords:** Flea beetles, leaf beetles, Malaise trap, new species, Shei-Pa National Park, taxonomy

## Abstract

Three new species of *Sphaeroderma* Stephens, 1831, *S.hsui***sp. nov.**, *S.changi***sp. nov.**, and *S.sheipaensis***sp. nov.** are described based on specimens from Shei-Pa National Park, Taiwan. A fourth new species, *S.jungchani***sp. nov.**, is described based on specimens from southern Taiwan. Delimitation of the genus is discussed based on *S.flavonotatum* Chûjô, 1937, which is redescribed, and the new species, *S.jungchani***sp. nov.**

## ﻿Introduction

The first species of *Sphaeroderma* was recorded from Taiwan by Chen (1934) as *S.apicalis* Baly, 1874. Three species were added by Chûjô (1937): *S.flavonotatum* Chûjô, *S.rubi* Chûjô and *S.tibiale* Chûjô. The fifth species was described as *S.postnigrum* Chûjô (Chûjô 1963). *Sphaerodermakondoi* Ohno, 1964, was the sixth species (Ohno 1964). *Sphaerodermachui* Kimoto, 1970, was the seventh and *S.babai* Chûjô, 1963 was added to the Taiwan fauna in the same paper (Kimoto 1970). The latter species was inadvertently included; it should be *S.postnigrum* Chûjô, 1963 (Kimoto and Chu 1996). Three more new species were described by Takizawa (1979): *S.varicolor* Takizawa, *S.alishanensis* Takizawa, and *S.nigroapicalis* Takizawa. In total, ten species have been described or recorded from Taiwan previously.

During a research project conducted by Dr Yu-Feng Hsu (徐堉峰) and myself entitled “A survey for selection of insect indicator species and their microhabitat usage in the Daxueshan area of Shei-Pa National Park”, six Malaise traps were set up at different altitudes ranging from 2620 to 3320 m. Two traps collected more than 60 specimens representing three species resembling oblong-bodied *Sphaeroderma*. Moreover, two of these have transverse antennal calli with well-developed supracallinal sulci, a character shared with members of *Sphaeroderma*. To redefine genus boundaries of *Sphaeroderma*, specimens of *S.flavonotatum* Chûjô and its allied species were studied. Both possess characteristic white spots on the elytra and are easily recognized members of Taiwan chrysomelid fauna.

## ﻿Material and methods

**Figure 1.**
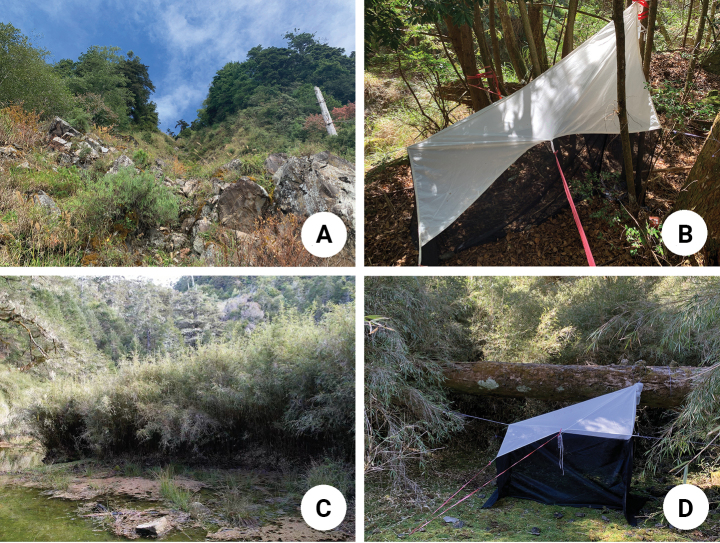
Habitat photographs **A** microhabitat at Hsishihshan trail (西勢山林道) **B** Malaise trap set up at Hsishihshan trail (西勢山林道) **C** microhabitat at Hsiaopangchih (小胖池) **D** Malaise trap set up at Hsiaopangchih (小胖池).

Five Malaise traps were set up at the Shei-Pa National Park from April 2021 to November 2022, namely Hsishihshan trail (Fig. 1B) (西勢山林道: 24°19'01.9"N, 121°03'36.3"E, 2630 m), Hsiaopangchih (Fig. 1D) (小胖池: 24°19'09.0"N, 121°04’09.7”E, 2830 m), Tahsuehshan (大雪山: 24°20'40.1"N, 121°07'36.2"E, 3280 m), Chichunshan (奇峻山: 24°20'58.8"N, 121°07'53.2"E, 3260 m), Tananshan (大南山: 24°21'40.3"N, 121°09'48.6"E, 3050 m), Huoshihshan (火石山: 24°22'47.8"N, 121°10'53.7"E, 3060 m). Adults of *Spheroderma* were collected from two localities, Hsishihshan trail and Hsiaopangchih. Microhabitats of both localities are described as follow:

Hsishihshan trail (Fig. 1A): plant cover composed of *Chamaecyparisformosensis* Matsum. (Cupressaceae) and *Pinustaiwanensis* Hayata (Pinaceae). Understory and herbaceous plants include *Illiciumanisatum* L. (Schisandraceae), *Photinianiitakayamensis* Hayata (Rosaceae), *Trochodendronaralioides* Siebold & Zucc. (Trochodendraceae), *Acerrubescens* Hayata (Sapindaceae), *Rhododendron* spp. (Ericaceae), *Euryacrenatifolia* (Yamam.) Kobuski and *E.glaberrima* Hayata (Pentaphylacaceae), *Symplocosmorrisonicola* Hayata (Symplocaceae), *Digitalispurpurea* L. (Plantaginaceae) and *Reynoutriajaponica* Houtt. (Polygonaceae).

Hsiaopangchih (Fig. 1C): a primeval forest with Tsugachinensis(Franch.)Pritz.var.formosana (Hayata) H.L. Li & H. Keng and *Abieskawakamii* (Hayata) T. Itô (Pinaceae) as dominant tree species. Minor tree species include *Pinusarmandii* Franch. var. mastersiana (Hayata) Hayata, *P.taiwanensis* (Pinaceae) and *Juniperussquamata* Lamb. (Cupressaceae). Understory shrubs include *Rhododendronformosanum* Hemsl. (Ericaceae), Juniperusformosanavar.formosana Hayata (Cupressaceae) and a shrub bamboo, *Yushanianiitakayamensis* (Hayata) Keng f. (Poaceae).

For taxonomic study, the abdomens of adults were separated from the forebodies and boiled in 10% NaOH solution, followed by washing in distilled water to prepare genitalia for illustrations. The genitalia were then dissected from the abdomens, mounted on slides in glycerin, and studied and drawn using a Nikon ECLIPSE 50i compound microscope with a drawing tube. Large morphological structures were drawn using a Leica M165 stereomicroscope with a drawing tube.

At least three males and females from each species were examined to delimit variability of diagnostic characters. For species collected from more than one locality or with color variations, at least one pair of each sex from each locality and color morph was examined. Length was measured from the anterior margin of the eye to the elytral apex, and width at the greatest width of the elytra. Nomenclature for morphological structures follows Duckett and Daza (2004).

Specimens studied herein are deposited at the Natural History Museum, London, UK (**BMNH**) and Applied Zoology Division, Taiwan Agricultural Research Institute, Taichung Taiwan (**TARI**).

Precise label data are cited for all type specimens of described species; a double slash (//) indicates label breaks and a single slash (/) indicates line breaks. Other comments and remarks are in square brackets: [p] – preceding data are printed, [h] – preceding data are handwritten, [w] – white label.

## ﻿Taxonomy

### ﻿ Sphaeroderma

Taxon classificationAnimaliaColeopteraChrysomelidae

Stephens, 1831

4BD579CD-BCBA-513A-9135-30AE163D7703

Sphaeroderma Stephens, 1831: 328. Type species: Alticatestacea Fabricius, 1775, subsequently designated by Maulik (1926).
Argosomus Wollaston, 1868: 152. Type species: Argosomusepilachnoides Wollaston, 1868, subsequently designated by Konstantinov and Vandenberg (1996). Synonymized by Scherer (1961).
Musaka Bechyné, 1958: 91. Type species: Sphaerodermafreyi Bechyné, 1955. Synonymized by Scherer (1961).
Kimotoa Gruey, 1985: 125. Type species: Argopussplendens Gressitt & Kimoto, 1963, by original designation. Synonymized by Konstantinov and Prathapan (2008).

#### Notes.

**Figure 2.**
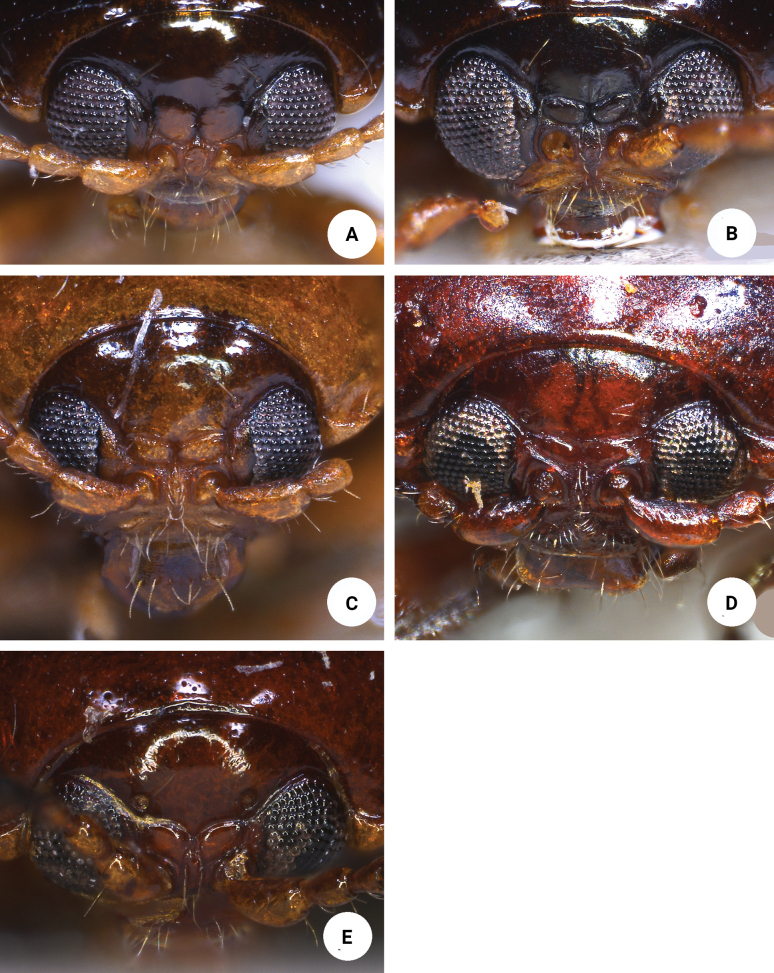
Heads of *Sphaeroderma* species in frontal view **A***S.hsui* sp. nov. **B***S.changi* sp. nov. **C***S.sheipaensis* sp. nov. **D***S.flavonotatum* Chûjô **E***S.jungchani* sp. nov.

**Figure 3.**
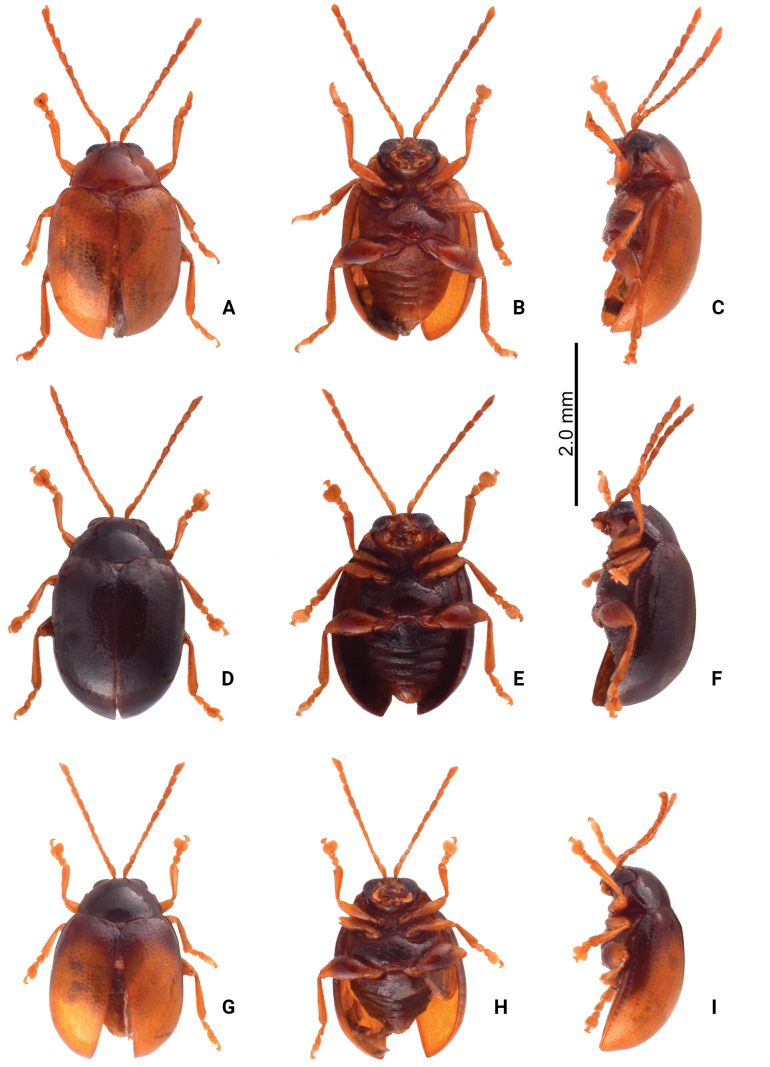
Habitus of *Sphaerodermahsui* sp. nov. **A** typical form, male, dorsal view **B** ditto, ventral view **C** ditto, lateral view **D** color variation, male, dorsal view **E** ditto, ventral view **F** ditto, lateral view **G** color variation, female, dorsal view **H** ditto, ventral view **I** ditto, lateral view.

Three new species with oblong bodies (Figs 3, 5) were collected from Shei-Pa National Park, but only one of them has longitudinal antennal calli (Fig. 2A) with poorly delimited supracallinal sulci. The other two have typical characters for *Sphaeroderma* (Fig. 2B–E). This suggests that the supracallinal sulci are not diagnostic.

**Figure 4.**
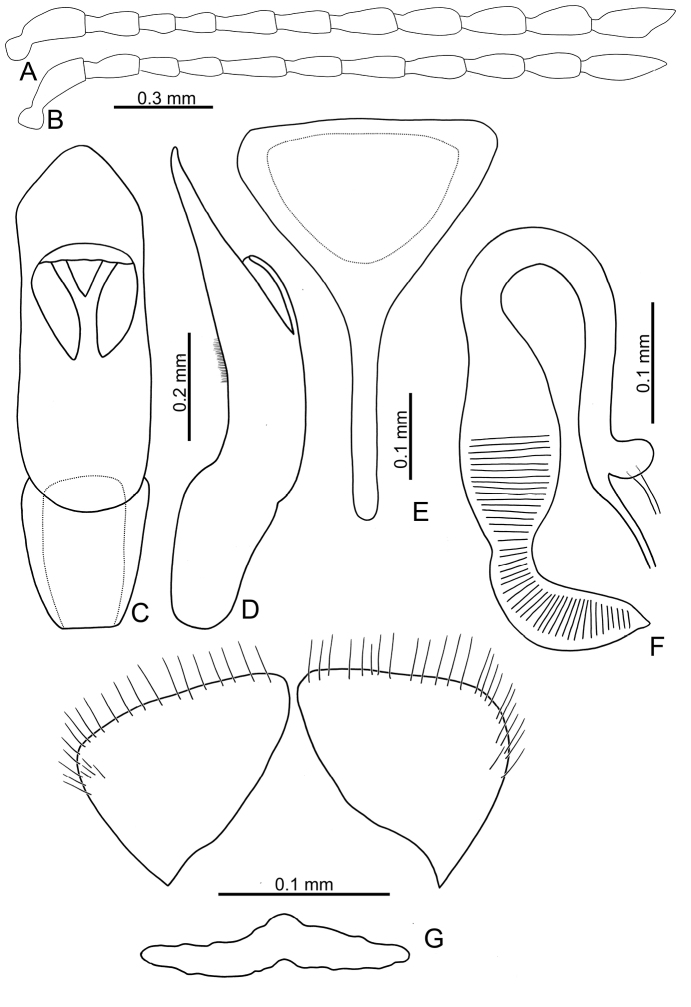
Diagnostic characters of *Sphaerodermahsui* sp. nov. **A** antenna, male **B** antenna, female **C** aedeagus, dorsal view **D** aedeagus, lateral view **E** abdominal ventrite VIII, female **F** spermatheca **G** gonocoxae.

**Figure 5.**
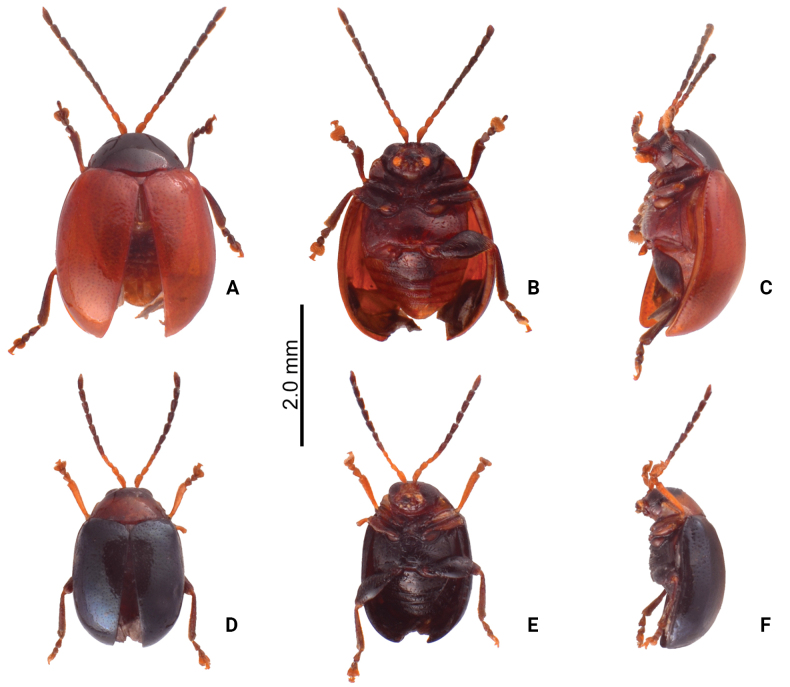
Habitus of *Sphaerodermachangi* sp. nov. and *S.sheipaensis* sp. nov. **A***S.changi* sp. nov., female, dorsal view **B** ditto, ventral view **C** ditto, lateral view **D***S.sheipaensis* sp. nov., female, dorsal view **E** ditto, ventral view **F** ditto, lateral view.

**Figure 6.**
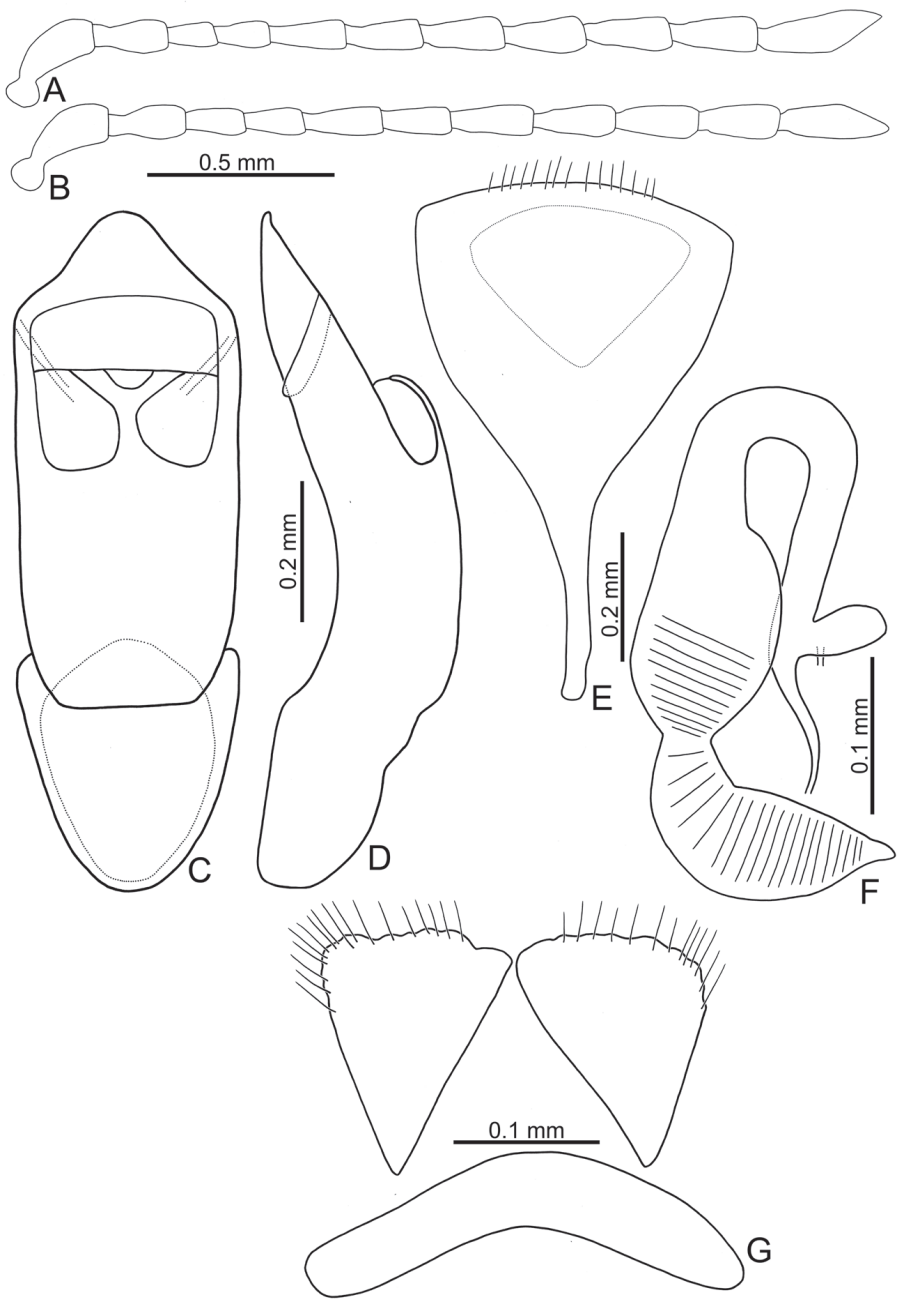
Diagnostic characters of *Sphaerodermachangi* sp. nov. **A** antenna, male **B** antenna, female **C** aedeagus, dorsal view **D** aedeagus, lateral view **E** abdominal ventrite VIII, female **F** spermatheca **G** gonocoxae.

**Figure 7.**
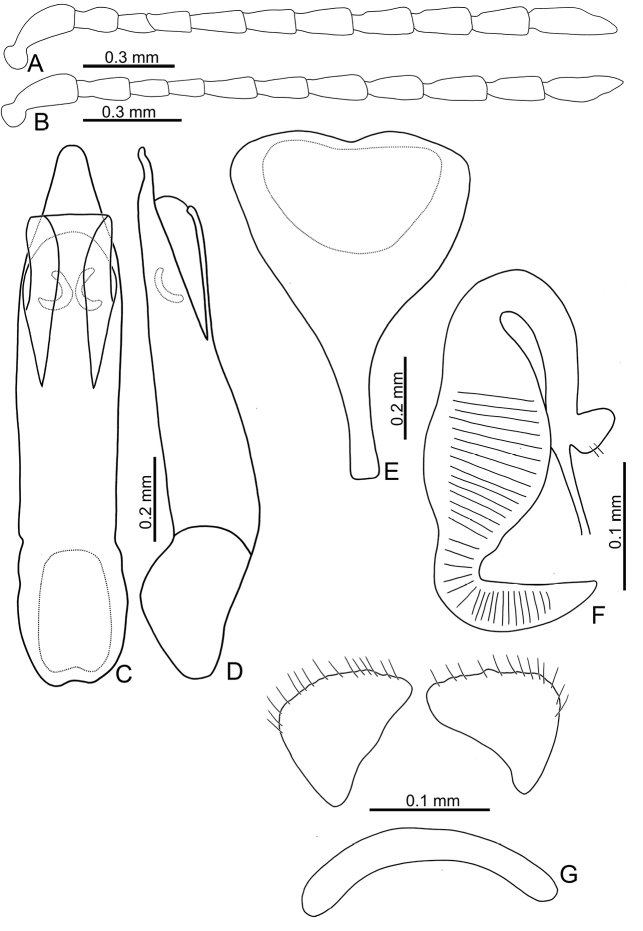
Diagnostic characters of *Sphaerodermasheipaensis* sp. nov. and *S.jungchani* Lee, sp. nov. **A** antenna, male, *S.jungchani* sp. nov. **B** antenna, female, *Sphaerodermasheipaensis* sp. nov. **C** aedeagus, dorsal view, *S.jungchani* sp. nov. **D** ditto, lateral view **E** abdominal ventrite VIII, female, *S.sheipaensis* sp. nov. **F** spermatheca, *S.sheipaensis* sp. nov. **G** gonocoxae, *S.sheipaensis* sp. nov.

**Figure 8.**
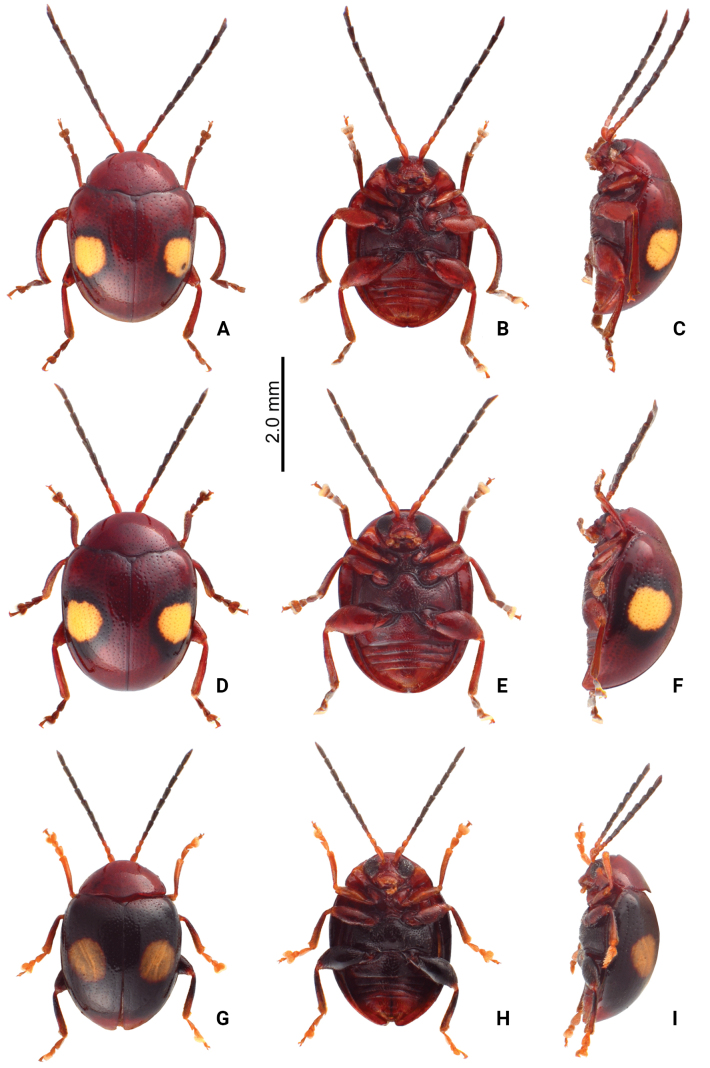
Habitus of *Sphaerodermaflavonotata* Chûjô and *S.jungchani* sp. nov. **A***S.flavonotata* Chûjô, male, dorsal view **B** ditto, ventral view **C** ditto, lateral view **D***S.flavonotata* Chûjô, female, dorsal view **E** ditto, ventral view **F** ditto, lateral view **G***S.jungchani* sp. nov., male, dorsal view **H** ditto, ventral view **I** ditto, lateral view.

**Figure 9.**
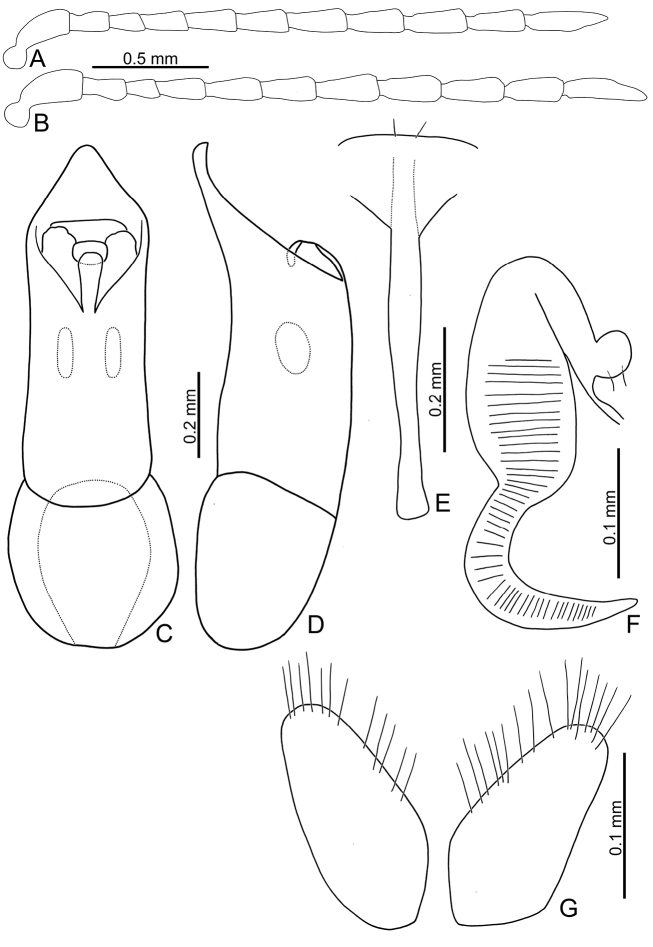
Diagnostic characters of *Sphaerodermaflavonotata* Chûjô **A** antenna, male **B** antenna, female **C** aedeagus, dorsal view **D** aedeagus, lateral view **E** abdominal ventrite VIII, female **F** spermatheca **G** gonocoxae.

The new species of *Sphaeroderma* from Shei-Pa National Park can be assigned to a species group (= *S.hsui* species group) that can be separated from other species of *Sphaeroderma* (such as *S.flavonotatum* and *S.jungchani* sp. nov.) by their oblong bodies (Figs 3, 5) which look like members of *Meishania* Chen & Wang, 1980: 1.1–1.2× longer and wide [spherical bodies (Fig. 8), as long as wide in *S.flavonotatum* and *S.jungchani* sp. nov.]; abdominal ventrites V without internal median ridge in males (abdominal ventrites V with internal median ridge in males of *S.flavonotatum* and *S.jungchani* sp. nov.), aedeagus without endophallic sclerites (Figs 4C, D, 6C, D) [aedeagus with one pair of small endophallic sclerites in *S.flavonotatum* (Fig. 9C, D) and *S.jungchani* sp. nov. (Fig. 7C, D)], gonocoxae with a transverse basal sclerite connected with apical sclerites (Figs 4G, 6G, 7G) [gonocoxae with only apical sclerites in *S.flavonotatum* (Fig. 9G)], and abdominal ventrite VIII in females strongly sclerotized and short speculum (Figs 4E, 6E, 7E) [abdominal ventrite VIII in females membranous except apical margin scleritozed and long speculum in *S.flavonotatum* (Fig. 9E)].

### ﻿ Sphaeroderma hsui sp. nov.

Taxon classificationAnimaliaColeopteraChrysomelidae

370802DE-1894-541F-8F5F-500876CFFF2F

https://zoobank.org/510555F9-6ABE-4D34-9789-0D27E498F030

Figs 2A , 3 , 4

#### Types.

***Holotype*** ♂ (TARI). Taiwan: Miaoli, Hsiaopangchih (小胖池), 28.VIII.2021–16.XI.2021, leg. Y.-F. Hsu. ***Paratypes*.** 29♂, 22♀ (TARI: 26♂, 19♀; BMNH: 3♂, 3♀), same data as holotype; 4♂, 2♀ (TARI), same but with “28.VIII.2021”; 2♂, 3♀ (TARI), same but with “21.V.-28.VIII.2021”; 1♀ (TARI): Hsishihshan (西勢山), 16.XI.2021–21.IV.2022, leg. Y.-F. Hsu.

#### Description.

Length 2.5–2.8 mm, width 1.6–1.9 mm. Body color (Fig. 3A–C) yellowish-brown. Antennae filiform in males (Fig. 4A), antennomeres VIII–X wider, length ratios of antennomeres I–XI 1.0:0.6:0.4:0.5:0.7:0.6:0.7:0.8:0.7:0.7:1.0, length to width ratios of antennomeres I–XI 2.9:2.1:1.8:2.0:2.5:2.4:2.5:2.6:2.3:2.3:3.0; similar shape in females (Fig. 4B), but antennomeres VIII–X narrower, length ratios of antennomeres I–XI 1.0:0.6:0.4:0.5:0.8:0.6:0.8:0.7:0.7:0.7:1.0, length to width ratios of antennomeres I–XI 3.1:2.3:1.9:2.2:3.2:2.6:2.9:2.5:2.4:2.2:3.3. Antennal calli longitudinal, with supracallinal sulci poorly delimited. Pronotum 1.7–1.9× wider than long, disc with fine, scattered punctures, same size as punctures on elytra; lateral margins almost straight; anterolateral callosity protruding forward; posterolateral callosity poorly developed. Elytra 1.3 times longer than wide, sides widely rounded; disc with punctures entirely confused; humeral calli well developed. Abdominal ventrite V without internal median ridge in both sexes. Male genitalia: aedeagus (Fig. 4C, D) slender in dorsal view, 4.0× longer than wide; parallel-sided, but slightly narrowed at apical 1/4, apex widely rounded; moderately curved at middle in lateral view, apex slightly recurved, ventral margin with densely, tiny teeth at middle; ostium membranous and with Y-shaped sclerotized area. Female genitalia: ventrite VIII (Fig. 4E) with apical part triangular, apical margin smooth and lacking setae, disc with medial part membranous, spiculum short; gonocoxae (Fig. 4G) separated, transversely triangular, with dense, long setae along apical margins, with one slender and transverse basal sclerite; receptacle of spermatheca (Fig. 4F) moderately swollen; pump short and strongly curved, transverse wrinkles present on entire pump and extending into half of receptacle; sclerotized proximal spermathecal duct long, with ramus oblong.

#### Color variation.

Some adults have black bodies (Fig. 3D–F) with legs and antennae that are yellowish-brown, with metafemora darkener; some adults (Fig. 3G–I) similar in this color form, but the elytra are yellowish-brown except darker base that extends to lateral margins, then abbreviated at apical 1/3.

#### Diagnosis.

Although adults of *Sphaerodermahsui* sp. nov. display diverse color patterns (Fig. 3), they are characteristic and diagnostic. In addition, this new species differs from the two other Taiwanese species (*S.changi* sp. nov. and *S.sheipaensis* sp. nov.) based the following combination characters: entire yellowish-brown antennae (Fig. 3) [yellowish-brown antennomeres I–IV and black antennomeres V–XI in other species (Fig. 5)]; longitudinal antennal calli with poorly delimited supracallinal sulci (Fig. 2A) [transverse antennal calli with well-developed supracallinal sulci in *S.changi* sp. nov. (Fig. 2B) and *S.sheipaensis* sp. nov. (Fig. 2C)]; slender aedeagus, 4.0× longer than wide, with cluster of setae at middle of inner margin in lateral view (Fig. 4D) [wide aedeagus, 3.1× longer than wide, and inner margin lacking setae in lateral view in *S.changi* sp. nov. (Fig. 6D)]; triangular abdominal ventrite VIII in females with apical margin lacking setae, subapically and moderately narrowed sides (Fig. 4E) [apical margin with seven pairs of setae at medial part in *S.changi* sp. nov. (Fig. 6E), subapically and slightly narrowed sides in *S.changi* sp. nov. and *S.sheipaensis* sp. nov. (Figs 6E, 7E)]; transversely triangular gonocoxae (Fig. 4G) [longitudinally triangular gonocoxae in *S.changi* sp. nov. (Fig. 6G)].

#### Etymology.

The name is dedicated to Dr Yu-Feng Hsu (徐堉峰), who is the director for the insect survey project at Shei-Pa National Park.

#### Distribution.

*Sphaerodermahsui* sp. nov. seems to be the dominant species at the Shei-Pa National Park.

### ﻿ Sphaeroderma changi sp. nov.

Taxon classificationAnimaliaColeopteraChrysomelidae

F967FCF8-5766-5534-A1D0-DA5F559F0B5D

https://zoobank.org/880B2056-1C8E-4C62-AFBB-434F07506557

Figs 2B , 5A–C , 6

#### Types.

***Holotype*** ♂ (TARI). Taiwan: Miaoli, Hsishihshan trail (西勢山林道), 26.IX.2021, leg. Y.-F. Hsu. ***Paratypes*.** 1♀ (TARI), same but with “28.VIII.2021”.

#### Description.

Length 3.2 mm, width 2.2 mm. Body color (Fig. 5A–C) yellowish or reddish-brown; head, pronotum, and legs blackish-brown; antennae blackish-brown except four basal antennomeres yellowish-brown. Antennae filiform in males (Fig. 6A), length ratios of antennomeres I–XI 1.0:0.7:0.4:0.5:0.7:0.7:0.8:0.8:0.8:0.8:1.2, length to width ratios of antennomeres I–XI 2.8:2.2:1.9:2.0:2.7:2.5:2.5:2.3:2.3:2.4:3.5; similar in females (Fig. 6B), length ratios of antennomeres I–XI 1.0:0.6:0.5:0.5:0.7:0.6:0.7:0.7:0.7:0.7:0.9, length to width ratios of antennomeres I–XI 2.9:2.2:2.1:2.1:2.7:2.4:2.7:2.6:2.5:2.4:3.2. Antennal calli transverse, with supracallinal sulci well developed. Pronotum 1.7× wider than long, disc with fine, scattered punctures the same size as those on elytra; lateral margins almost straight; anterolateral callosity protruding forward; posterolateral callosity poorly developed. Elytra 1.1–1.2 times longer than wide, sides widely rounded; disc with punctures entirely confused; humeral calli well developed. Abdominal ventrite V without internal median ridge in both sexes. Male genitalia: aedeagus (Fig. 6C, D) wide in dorsal view, 3.1× longer than wide; parallel-sided, apex narrowly rounded, strongly widened at apical 1/8; moderately curved at middle in lateral view; ventral surface with one pair of oblique ridges from apical 1/5 of lateral margins; ostium membranous and with Y-shaped sclerotized area. Female genitalia: ventrite VIII (Fig. 6E) with apical part triangular, but sides slightly narrowed near apex, apical margin smooth and with seven pairs of setae medially, disc with medial part membranous, spiculum short; gonocoxae (Fig. 6G) separated, longitudinally triangular, with dense, long setae along apical margins, with one slender and transverse basal sclerite; receptacle of spermatheca (Fig. 6F) moderately swollen; pump short and strongly curved, transverse wrinkles present on entire pump and extending onto half of receptacle; sclerotized proximal spermathecal duct long, with ramus oblong.

#### Diagnosis.

Adults of *Sphaerodermachangi* sp. nov. have a characteristic and diagnostic color pattern (Fig. 5A–C). In addition, this new species differs from the other two Taiwanese species (*S.hsui* sp. nov. and *S.sheipaensis* sp. nov.) based on the following combination of characters: yellowish-brown antennomeres I–IV and black antennomeres V–XI (Fig. 5A–C) [entire yellowish-brown antennae in *S.hsui* sp. nov. (Fig. 3)]; transverse antennal calli with well-developed supracallinal sulci (Fig. 2B) [longitudinal antennal calli with poorly delimited supracallinal sulci in *S.hsui* sp. nov. (Fig. 2A)]; wide aedeagus, 3.1× longer than wide, ventral disc with one pair of oblique ridges from apical 1/5 in lateral margin (Fig. 6C, D) [slender aedeagus, 4.0× longer than wide and ventral disc without oblique ridges in *S.hsui* sp. nov. (Fig. 4C, D)]; triangular abdominal ventrite VIII in females with apical margin with seven pairs of setae at medial part, subapically and slightly narrowed sides (Fig. 6E) [apical margin lacking setae; subapically and moderately narrowed sides in *S.hsui* sp. nov. (Fig. 4E)]; longitudinally triangular gonocoxae (Fig. 6G) [transversely triangular gonocoxae in *S.hsui* sp. nov. (Fig. 4G) and *S.sheipaensis* sp. nov. (Fig. 7G)].

#### Etymology.

This species is named for Mr Li-Jen Chang (張勵仁) for his assistance in conducting the project.

#### Distribution.

*Sphaerodermachangi* sp. nov. is a rarely collected species known from only one locality in Shei-Pa National Park.

### ﻿ Sphaeroderma sheipaensis sp. nov.

Taxon classificationAnimaliaColeopteraChrysomelidae

01778E7E-1179-5F4D-A55D-5C81E40569CD

https://zoobank.org/08FADE40-DBF7-44CB-BDFD-4B08D0138BF1

Figs 3C , 5D–F , 7B, E–G

#### Types.

***Holotype*** ♀ (TARI). Taiwan: Miaoli, Hsishihshan trail (西勢山林道), 28.VIII.2021, leg. Y.-F. Hsu. ***Paratype*.** 1♀ (TARI), Hsiaopangchih (小胖池), 28.VIII.2021, leg. Y.-F. Hsu.

#### Description.

Length 2.4 mm, width 1.8 mm. Body color (Fig. 5D–F) blackish-brown, head, prothorax, scutellum yellowish-brown; elytra metallic blue, metafemora blackish-brown; antenna black except four basal antennomeres yellowish-brown. Antennae filiform in females (Fig. 7B), length ratios of antennomeres I–XI 1.0:0.6:0.4:0.4:0.6:0.6:0.7:0.7:0.8:0.7:1.0, length to width ratios of antennomeres I–XI 2.9:2.2:2.2:2.0:2.5:2.5:2.3:2.3:2.5:2.2:3.4. Antennal calli transverse, with supracallinal sulci well developed. Pronotum 1.9 times wider than long, disc with fine, scattered punctures the same size as those on elytra; lateral margins almost straight; anterolateral callosity protruding forward; posterolateral callosity poorly developed. Elytra 1.1 times longer than wide, sides widely rounded; disc with punctures entirely confused; humeral calli well developed. Female genitalia: ventrite VIII (Fig. 7E) with apical part triangular, but sides slightly narrowed near apex, apical margin smooth and depressed at middle, lacking setae; disc with medial part membranous, spiculum short; gonocoxae (Fig. 7G) separated, transversely triangular, with dense, long setae along apical margins, with one slender and transverse basal sclerite; receptacle of spermatheca (Fig. 7F) moderately swollen; pump short and strongly curved, transverse wrinkles present on entire pump and extending onto half of receptacle; sclerotized proximal spermathecal duct long, with ramus oblong.

**Males** unknown.

#### Diagnosis.

Adults of *Sphaerodermasheipaensis* sp. nov. have a characteristic and diagnostic color pattern (Fig. 5D–F). In addition, this new species differs from the two other Taiwanese species (*S.hsui* sp. nov. and *S.changi* sp. nov.) based on the following combination of characters: yellowish-brown antennomeres I–IV and black antennomeres V–XI (Fig. 5D–F) [entire yellowish-brown antennae in *S.hsui* sp. nov. (Fig. 3)]; transverse antennal calli with well-developed supracallinal sulci (Fig. 2C) [longitudinal antennal calli with poorly delimited supracallinal sulci in *S.hsui* sp. nov. (Fig. 2A)], triangular abdominal ventrite VIII in females with apical margin lacking setae, subapically and slightly narrowed sides (Fig. 7E) [apical margin with seven pairs of setae at medial part in *S.changi* sp. nov. (Fig. 6E), subapically and moderately narrowed sides in *S.hsui* sp. nov. (Fig. 4E)]; transversely triangular gonocoxae (Fig. 7G) [longitudinally triangular gonocoxae in *S.changi* sp. nov. (Fig. 6G)].

#### Etymology.

The species is named for Shei-Pa National Park (雪霸國家公園) where three new species were collected.

#### Distribution.

*Sphaerodermasheipaensis* sp. nov. is a rarely collected species known from two localities in Shei-Pa National Park.

### ﻿ Sphaeroderma flavonotatum

Taxon classificationAnimaliaColeopteraChrysomelidae

Chûjô, 1937

64923598-26ED-583D-ADEC-4AB1986FBD4E

Figs 8A–F , 9 , 10

Sphaeroderma flavonotata Chûjô, 1937: 40; Kimoto 1966: 35 (Hoozan = Fenghuangshan, 鳳凰山); Kimoto 1970: 295 (Fenchihu 奮起湖).

#### Types.

***Lectotype*** ♂ (TARI), here designated for clarifying its species identity which was confused with *S.jungchani* sp. nov., labeled: “Arisan (= Alishan, 阿里山) / 1912.X.10 [h] / Col. I. Nitobe [p, w] // Co / Type (p, circle label with yellow letters and border) // Sphaeroderma / flavonotata / Chûjô [h] / DET. M. CHUJO [p, w] // 1020 [p, w]”. Paralectotypes. 1♂, 3♀ (TARI), same data as holotype, but with “2683 (♂), 1388 (♀), 1917 (♀), 2684 (♀)”; 1♂: “Mt. Arisan / FORMOSA / 25.X.1933 [h] / COL. M CHUJO [p, w] // Co / Type (p, circle label with yellow letters and border) // Sphaeroderma / flavonotata / Chûjô [h] / DET. M. CHUJO [p, w] // 698 [p, w]”.

#### Other material.

Taiwan. Hsinchu: 2♂, 1♀ (TARI), Kuanhsi (關西), 2.IX.2011, leg. H. Lee; 2♂, 1♀ (TARI), Wuchihshan (五指山), 27.III.2008, leg. H. Lee; Nantou: 1♂ (BMNH), Tungpu (東埔), 5–8.X.1981, leg. T. Lin & W. S. Tang; 2♀ (BMNH), same but with “18–23.XI.1981”; 1♂ (BMNH)), same locality, 18–23.VII.1982, leg. L. Y. Chou & T. Lin; Pingtung: 1♂ (TARI), Tahanshan (大漢山), 7.II.2008, leg. M.-H. Tsou; 1♀ (TARI), same but with “leg. S.-F. Yu”; 1♀ (TARI), same locality, 3.III.2008, leg. C.-F. Lee; Taichung: 1♂, 1♀ (TARI), Wushihkeng (烏石坑), 19.III.2008, leg. C.-F. Lee; Taipei: 1♀ (TARI), Fushan (福山), 15.III.2007, leg. H.-J. Chen.

#### Redescription.

Length 3.1–3.5 mm, width 2.2–2.5 mm. Body color (Fig. 8A–F) reddish-brown; antennae blackish-brown except three basal antennomeres reddish-brown; elytron with one large white, round spot, margined by black, black area variable in extent; legs sometimes darker. Antennae filiform in males (Fig. 9A), apex of antennomere III angular, much smaller than IV, length ratios of antennomeres I–XI 1.0:0.5:0.4:0.6:0.7:0.7:0.8:0.8:0.8:0.8:1.1, length to width ratios of antennomeres I–XI 3.1:2.0:1.8:2.3:2.3:2.2:2.6:2.5:2.6:2.6:4.3; similar shape in females (Fig. 9B), length ratios of antennomeres I–XI 1.0:0.5:0.3:0.6:0.6:0.6:0.7:0.7:0.7:0.7:1.0, length to width ratios of antennomeres I–XI 2.8:2.1:1.7:2.3:2.4:2.2:2.3:2.4:2.5:2.4:4.1. Antennal calli transverse, with supracallinal sulci well developed. Pronotum 1.6–1.7× wider than long, disc with coarse, scattered punctures, mixed with fine punctures; lateral margins slightly rounded; anterolateral callosity protruding forward; posterolateral callosity poorly developed. Elytra as long as wide, sides widely rounded; disc with punctures arranged into longitudinal striae but mixed with additional punctures; humeral calli well developed. Middle tibiae curved in males (Fig. 8A, B), and much longer than other tibiae. Abdominal ventrite V with median internal ridge in males. Male genitalia: aedeagus (Fig. 9C, D) wide in dorsal view, 3.6× longer than wide; parallel-sided, apex narrowly rounded, strongly widened at apical 1/6; slightly curved at middle in lateral view, extremely wide; ostium membranous and with short median longitudinal sclerite, and with one rounded sclerite connected with apex; internal sac with one pair of rounded and laterally flattened sclerites. Female genitalia: ventrite VIII (Fig. 9E) membranous, only apical margin sclerotized, with one pair of setae near middle, spiculum long; gonocoxae (Fig. 9G) separated, oblong, with dense, long setae along apical margins; receptacle of spermatheca (Fig. 9F) moderately swollen; pump long and strongly curved, transverse wrinkles present on entire pump and extending onto half of receptacle; sclerotized proximal spermathecal duct long, with ramus rounded.

#### Diagnosis.

Adults of *S.flavonotatum* Chûjô and *S.jungchani* sp. nov. are easily separated by the color surrounding the single pair of large white spots on the elytra: black margins on reddish-brown elytra in *S.flavonotatum*; reddish-brown surrounding spots in *S.jungchani* sp. nov. Middle tibiae are sexually dimorphic, enlarged in males of *S.flavonotatum* (middle tibiae not modified in males of *S.jungchani* sp. nov.). The aedeagus is broader in *S.flavonotatum*, 3.6× longer than wide, and thick in lateral view (Fig. 9C, D) [narrow aedeagus, 5.2× longer than wide, and thin in lateral view in *S.jungchani* sp. nov. (Fig. 7C, D)].

#### Host plants.

Clematistashiroivar.tashiroi Maxim. (Ranunculaceae) (Lee and Cheng 2010).

#### Distribution.

**Figure 10.**
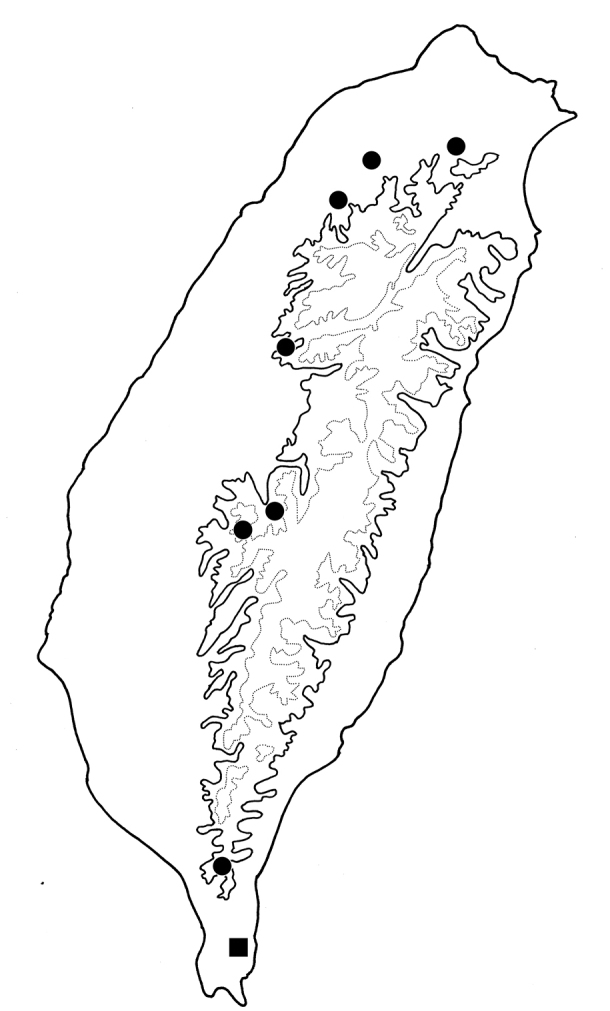
Distribution map of *Sphaerodermaflavonotata* Chûjô and *S.jungchani* sp. nov. Solid line: 1000 m, dotted line: 2000 m; circles: *S.flavonotata* Chûjô; square: *S.jungchani* sp. nov.

Widespread in Taiwan (Fig. 10).

### ﻿ Sphaeroderma jungchani sp. nov.

Taxon classificationAnimaliaColeopteraChrysomelidae

9F40AA65-E1D5-5937-B345-A52622FFBD56

https://zoobank.org/AB83115A-5632-466C-B5B5-401A9B92A089

Figs 7A, C, D ; 8G–I , 10

#### Types.

***Holotype*** ♂ (TARI). Taiwan: Pingtung, Nanjenhu (南仁湖), 31.III.2011, leg. J.-C. Chen. ***Paratypes*.** 3♂ (BMNH: 2♂; TARI: 1♂), same data as holotype; 1♂ (TARI), same but with “11.IV.2011”; 1♂ (TARI), same but with “3.IV.2023”.

#### Description.

Length 2.9-3.3 mm, width 2.1–2.3 mm. Body color (Fig. 8G–I) blackish-brown; head and prothorax reddish-brown; antennae blackish-brown except three basal antennomeres reddish-brown; elytron with one large white round spot, elytra basally and apically reddish-brown; abdominal ventrite V reddish-brown; legs dark brown but front legs paler. Antennae filiform in males (Fig. 7A), apex of antennomere III angular, much smaller than IV, length ratios of antennomeres I–XI 1.0:0.5:0.4:0.5:0.6:0.6:0.7:0.7:0.7:0.7:1.1, length to width ratios of antennomeres I–XI 2.9:1.7:1.6:2.1:2.3:2.1:2.1:2.2:2.1:2.1:3.4. Pronotum 1.7–1.8× wider than long, disc with coarse, scattered punctures, mixed with fine punctures; lateral margins slightly rounded; anterolateral callosity protruding forward; posterolateral callosity poorly developed. Elytra 1.1–1.2 times longer than wide, sides widely rounded; disc with punctures arranged into longitudinal striae but mixed with additional punctures; humeral calli well developed. Abdominal ventrite V with median internal ridge in males. Antennal calli transverse, with supracallinal sulci well developed. Male genitalia: aedeagus (Fig. 7C, D) narrow in dorsal view, 5.2× longer than wide; parallel-sided, apex narrowly rounded, strongly widened at apical 1/6; almost straight at middle in lateral view and narrow, apex sinuate; ostium membranous and with long median longitudinal sclerite; internal sac with one pair of curved sclerites.

**Females** unknown.

#### Diagnosis.

Adults of *S.jungchani* sp. nov. and *S.flavonotatum* Chûjô are easily separated by the color surrounding the single pair of large white spots on the elytra: surrounded by reddish-brown elytra in *S.jungchani*; black borders surrounding spots on reddish-brown elytra of *S.flavonotatum* sp. nov. Middle tibiae of *S.jungchani* sp. nov. males not modified (sexually dimorphic middle tibiae in *S.flavonotatum*). Aedeagus narrow in *S.jungchani* sp. nov., 5.2× longer than wide, and thin in lateral view (Fig. 7C, D) [aedeagus broad, 3.6× longer than wide, and thick in lateral view in *S.flavonotatum* (Fig. 9C, D)].

#### Host plants.

*Chloranthusoldhamii* Solms (Chloranthaceae).

#### Etymology.

The species is named for Mr Jung-Chan Chen (陳榮章) for collecting type specimens of this new species.

#### Distribution.

Only known in type locality, Nanjenhu (南仁湖), in southern Taiwan (Fig. 10). This new species is allopatric from *S.flavonotatum*.

## ﻿Discussion

The current study suggests that adults of the *Sphaerodermahsui* species group can be collected efficiently with Malaise traps set up at suitable microhabitats. No specimens in this group were collected above 3000 m at the Shei-Pa National Park, central Taiwan (with three traps). Members of the group may be limited to 2600–3000 m, where other *Sphaeroderma* species are absent. Moreover, the three species were collected from a single national park. The diversity of this species group in Taiwan should be greater than current knowledge suggests.

Color patterns were used as key characters for diagnosis of Taiwanese species of *Sphaeroderma* (Kimoto and Takizawa 1997). This study suggests that color variation will be documented in species when a sufficient number of specimens become available for study. Thus, a revision of this genus based on more material is needed.

The genus *Meishania* was erected by Chen and Wang (1980) for the single species, *M.rufa* Chen & Wang, from Sichuan, China. Five more species were added to the genus from Sichuan and Yunnan (China), and Bhutan by Ruan et al. (2018). Members of *Meishania* have broad, entire third tarsomeres, a character state shared with *Argopistes* Motschulsky, 1860, *Argopus* Fischer von Waldheim, 1824, *Bhamoina* Bechyné, 1958, *Chilocoristes* Weise, 1895, *Jacobyana* Maulik, 1926, *Omeisphaera* Chen & Zia, 1974, *Parargopus* Chen, 1939, *Pentamesa* Harold, 1876 and *Sphaeroderma* Stephens, 1831. All of these genera can be separated from *Meishania* by the hemispherical body. Ruan et al. (2018) suggested that specimens of *Meishania* are closely allied to those of *Sphaeroderma*, but could be recognized by their oblong bodies (spherical bodies in *Sphaeroderma*), longitudinal antennal calli with poorly delimited supracallinal sulci (transverse antennal calli with well-developed supracallinal sulci in *Sphaeroderma*). Adults of *Sphaeroderma*, were characterized as having spherical bodies with transverse antennal calli and well-developed supracallinal sulci. Three new species, described here, with oblong bodies (Figs 3, 5) were collected from Shei-Pa National Park. One of them, *Sphaerodermahsui* sp. nov., possesses longitudinal antennal calli (Fig. 2A) with poorly delimited supracallinal sulci. The other two have typical characters for *Sphaeroderma* (Figs 2B–E). Delimitation of both *Sphaeroderma* and *Meishania* requires further study because these overlapping characters obscure generic boundaries.

## Supplementary Material

XML Treatment for Sphaeroderma

XML Treatment for Sphaeroderma hsui

XML Treatment for Sphaeroderma changi

XML Treatment for Sphaeroderma sheipaensis

XML Treatment for Sphaeroderma flavonotatum

XML Treatment for Sphaeroderma jungchani

## ﻿Additional information

### Author ORCIDs

Chi-Feng Lee https://orcid.org/0000-0003-1996-0557

### Data availability

All of the data that support the findings of this study are available in the main text.
